# Mental health problems, health risk behaviors, and prevention: A qualitative interview study on perceptions and attitudes among elite male soccer players

**DOI:** 10.3389/fpubh.2022.1044601

**Published:** 2023-01-05

**Authors:** Pia Kvillemo, Anders Nilsson, Anna K. Strandberg, Karl Björk, Tobias H. Elgán, Johanna Gripenberg

**Affiliations:** ^1^STAD, Centre for Psychiatry Research, Department of Clinical Neuroscience, Stockholm Health Care Services, Region Stockholm, Karolinska Institutet, Stockholm, Sweden; ^2^Centre for Psychiatry Research, Department of Clinical Neuroscience, Stockholm Health Care Services, Region Stockholm, Karolinska Institutet, Stockholm, Sweden; ^3^Stockholm Health Care Services, Region Stockholm, Karolinska Universitetssjukhuset, Stockholm, Sweden

**Keywords:** gambling, football, alcohol, substance use, elite athletes, policy work, risk and protective factors

## Abstract

**Background:**

The purpose of this study was to investigate the perception of mental health problems and health risk behaviors among Swedish male elite soccer players and their attitudes toward possible prevention strategies.

**Method:**

Twenty elite soccer players, aged 15–30 years, were recruited through purposive sampling and interviewed *via* a digital video calling platform. A semi-structured interview guide, encompassing questions about mental health problems, health risk behaviors among soccer teams, peer-relations, relations to coaches, and attitudes toward health risk behaviors, along with proposals for effective interventions, was employed. The interviews were transcribed verbatim and analyzed with qualitative content analysis.

**Results:**

The informants reported positive feelings in relation to playing soccer, good health, and few health risk behaviors. Risk factors included a large income, excessive free time, and the need for excitement. Stress and mental health problems were linked to performance pressure, social media, and injuries. Hesitation to talk openly about personal problems due to concerns about negative consequences and the “macho culture” was highlighted as barriers to admit and seek help for personal problems. Some statements indicated openness and the club's efforts to destigmatize personal problems. Positive attitudes toward prevention and suggestions for various measures were prominent.

**Conclusion:**

Future research and implementation of interventions should focus on the prevention of health risk behaviors and alleviation of stress and performance pressure, as well as continue the efforts to destigmatize mental health problems and raise awareness among coaches of the importance of their communication and behavior for players' mental health and performance. This could be achieved by developing strategic and systematic policy work, information, and dialogue among players and coaches, in addition to individual digital or face-to-face support, provided by professionals outside the soccer context.

## 1. Background

The attention to health among professional soccer players has traditionally focused on physical condition and injuries, with interventions directed to prevention and recovery (1–3). In recent decades, the interest in mental health among elite athletes, including soccer players has increased (1–5). Elite athletes are exposed to stress-related risk factors such as constant pressure to perform, overtraining, tissue damages, extensive traveling, and challenges in family and social life, which may result in health risk behaviors as well as mental health problems (1, 2, 5–10).

A growing number of epidemiological studies have addressed the prevalence of mental health problems in elite athletes, with varying results. For example, the reported prevalence of mental health disorders (11) and mental health symptoms among male elite athletes in team sports (cricket, soccer, handball, ice hockey, and rugby) has been found to vary from 5% for burnout and adverse alcohol use to nearly 45% for anxiety and depression (10). In one of the first studies on distress, anxiety/depression, and substance abuse/dependence among professional soccer players (*n* = 540), Gouttebarge et al. (4) included players from five European countries (Finland, France, Norway, Spain, and Sweden). The highest observed 1-month prevalence or point prevalence of symptoms was 18% for distress (Sweden), 43% for anxiety/depression (Norway), 33% for sleeping disturbance (Spain), 17% for adverse alcohol behavior (Finland), and 74% for adverse nutrition behavior (Norway). However, these figures should be interpreted with caution due to the limited sample sizes and varying response rates among the nation-based samples. Along with other addictive behaviors, gambling among elite soccer players has raised concern in recent years (9, 12–14). Problem gambling has been found to be associated with the competitive and emotional challenges of gambling (capturing the thrill and euphoria associated with competitive success on the field, or gambling to alleviate negative feelings), high salaries, spare time, and the social networks of young soccer players (13). Comparing the current figures of health risk behaviors and mental health status among elite soccer players with corresponding estimates in the general population is difficult for a number of reasons (5). Reardon et al. (10) have pointed out the following obstacles to such comparisons: (a) many studies of elite athletes' mental health do not include a general population reference group, (b) different instruments are used to assess mental health among athletes compared to the general population, (c) cultural differences in meanings and manifestations of mental health problems may complicate the interpretation of results, and (d) variation with regard to the type of data in the studies (e.g., self-reported or physician diagnosed disorders). Interpreting and comparing results from research on the mental health of elite athletes is also challenged by differences in the measurements chosen, risk of participation bias, and low response rates (4, 8, 9). Therefore, investigating mental health problems among elite athletes with qualitative studies is vital to build a greater knowledge base about mental health in sports, from which, interventions can be practically applied, adapted, or evaluated, and the impact of this research on practice can be increased (15).

The association between mental health and athletic performance in soccer and the importance of the players' health to obtain victories for their teams and clubs (16, 17) makes mental health vital to soccer leagues around the world, and a number of initiatives to prevent mental health problems have been implemented (10, 18, 19). Interventions to prevent mental health problems and health risk behaviors among elite athletes encompass efforts to create a supportive environment with policy work regarding inclusion, communication, strengthening of intrinsic motivation, and regulations around health risk behaviors, as well as interventions directed to the individual athlete, such as self-management training and promotion of adaptive coping strategies (16, 20). The concept “mental health” has historically been defined in different ways. In the current study the authors define mental health in accordance with the World Health Organization as “a state of complete physical, social, and mental wellbeing, and not merely the absence of disease or infirmity” (21). Using the classification system of the American Psychiatric Association, the authors of this study define mental disorders as “syndromes characterized by clinically significant disturbance in an individual's cognition, emotional regulation, or behavior that reflects a dysfunction in the psychological, biological, or developmental processes underlying mental functioning”, including substance use disorder and gambling disorder (22). Further, the authors of this study define “health risk behavior” as “an activity carried out by people with a frequency or intensity that increases the risk of disease or injury” (23), such as physical inactivity, unhealthy diet, or substance use (24).

Research and interventions in the elite soccer context are to a large extent focused on adult players (25), despite the many junior players that must also handle a competitive environment. Because prevention interventions at an early age may produce the greatest impact on health and wellbeing, addressing younger soccer players is crucial (26). The Swedish Professional Soccer Leagues (SEF), which organizes male senior and junior elite soccer clubs in Sweden, has in collaboration with our research group, initiated a project on mental health among male soccer players in 32 clubs in the 2 top divisions with the long-term aim of developing effective prevention interventions targeting mental health problems and various health risk behaviors (27). The current qualitative study is part of this project, which is why only male athletes are included. The project is informed by theories of addressing risk factors as well as protective factors for improving health (28), as a number of potential protective factors, including self-determined motivation, positive relations in sports and private life, and adequate recovery, have been found in elite athlete contexts (1, 16, 29). Furthermore, the project relies on the assumption that most human behavior is based on intentions (30), originating from attitudes influenced by personal knowledge, abilities, and social context (31–34). The exploration of elite soccer players' perception of mental health problems and health risk behaviors in the elite soccer context, as well as their attitudes toward ways of addressing these issues, is important for preventing mental health problems and promoting athletic performance at the elite level.

Few studies have examined the mental health experiences of elite athletes in parallel with their experiences or views on mental health support. The only study that the authors of this study found explored UK male professional cricketers' mental health and mental health support experiences (35). Ogden et al. (35) detected three key themes regarding mental health or mental health problems: (1) the emotional rollercoaster of a career in professional cricket, (2) local vs. national mental health support, and (3) nourishing vs. malnourishing players' mental health. Understanding mental health disorder prevalence, barriers to support seeking, mental toughness, and psychiatric epidemiology, are important for understanding the broader picture of mental health research and to further strengthen work undertaken in this area (5). To the best of our knowledge, this is the first study to qualitatively investigate junior and senior elite soccer players' views on mental health problems and health risk behaviors in the elite soccer context in parallel with their attitudes toward possible prevention interventions in Sweden. Because soccer is the most popular sport in the world (36) and players' wellbeing is positively associated with good performance (16, 17), the current study will make a valuable contribution to the literature regarding the prevention of mental health problems among elite athletes and soccer players, in particular. The authors specifically addressed the following research questions:

- How do professional soccer players perceive the occurrence, reasons for, and consequences of mental health problems in the male elite soccer context?
- How do professional soccer players perceive the occurrence, reasons for, and consequences of health risk behaviors, such as gambling, gaming, alcohol consumption, and consumption of other substances in the male elite soccer context?
- What are professional soccer players' attitudes toward possible prevention strategies that could be implemented in the male elite soccer context?

## 2. Method

### 2.1. Study design

A qualitative interview study was carried out among Swedish adult and junior (i.e., boys 15–19 years old) elite soccer players. The current study is part of a larger study, encompassing surveys as well as these interviews among elite soccer players and staff, such as coaches and managers, working in soccer clubs in the two top divisions. The purpose of the larger study is to develop intervention strategies to prevent mental health problems and health risk behaviors in the elite soccer context.

### 2.2. Recruitment and procedure

Purposive sampling was used to recruit the informant group. Elite senior and junior players were invited to participate in the study *via* e-mail. Contact information for the players was acquired from The Swedish Professional Soccer Leagues, who reached out to the teams and asked them to provide players for the study. The teams were to select players with and without previous experience of problems related to mental health and other health risk behaviors. Before agreeing to participate, the players were informed of the aim of the study, that participation was voluntary and anonymous (except in relation to the interviewer), that the interviews were to be carried out by phone or a digital video calling platform, that collected data were to be stored safely without unauthorized access and compiled so that no single participant could be identified, and that they could discontinue participation at any time without explanation. Before starting the interviews, informed consent was obtained and recorded by asking the informant if he agreed to participate in the study. If potential informants refrained from participation, additional players were contacted until a sufficient number of informants was recruited (37). Leaders or staff in the clubs or league were not informed of which players signed up for participation.

### 2.3. Participants

The informant group included a total of 20 players: 12 juniors, aged 15–19 years, and 8 senior players, aged 26–30 years. Information on ethnicity or country of origin was not collected as it was not regarded as relevant. Background information about the informants' age, living/household situation, and occupation are presented in Table 1.

**Table 1:** Background information of the informants.

**Informant^a^**	**Age (yr)**	**Living/household situation**	**Occupation outside of soccer^b^**
1	15	Parents and sibling	Student
2	16	Teammate	Student
3	16	Alone	Student
4	16	Parents	Student
5	17	Parents and sibling	“Odd jobs”
6	17	Parents and siblings	Student
7	17	Parents and sibling	Student
8	17	Parents	Student
9	18	Parents and sibling	Student
10	18	Parents and siblings	Student
11	19	Partner	“Odd jobs”
12	19	Parents and sibling	Student
13	25	Alone	Soccer coach
14	26	Alone	None
15	29	Alone	None
16	29	Partner and children	Bank clerk
17	30	Partner	Social worker, student
18	30	Partner	Starting up own business
19	30	Partner and child	None
20	30	Partner	Car repairman

a The numbers in the column “Informant” are not corresponding to the numbers displayed in connection to the citations in the result section.

b “None”, in the column of occupation means that the player lives solely on the income from soccer. Having an occupation means that the player has other incomes too.

### 2.4. Interviews

The interviews were conducted in the Swedish language by four of the authors (AN, AKS, KB, and PK) *via* a digital video calling platform during March 2021- January 2022. A semi-structured interview guide with open ended questions, suggestions of probing statements and follow-up questions, developed by the research team, was used. AN and AKS conducted three interviews each, KB conducted nine interviews, and PK conducted five. The questions and probing statements addressed personal experiences as well as observed mental health problems and health risk behaviors in the soccer team, peer-relations, relations between coach and player, social situation/background information, and attitudes toward health risk behaviors and effective or desirable prevention interventions. After 20 interviews had been conducted, it was assessed that no or little new information could be obtained by additional interviews and the interview process was terminated (37). The interviews, on average 38 min long, were recorded on audio files and transcribed verbatim. Every transcription was given a number, corresponding to a list with e-mail addresses for the informants, i.e., key-code. The list was kept separated from the transcriptions. The study was performed in accordance with the Declaration of Helsinki and APA ethical standards, and was approved by the Ethical Review Authority, registration number 2020-05010. Participants gave written and oral consent to participate in the study.

### 2.5. Transparency and openness

Data were structured in Nvivo 12, which facilitated the analysis process. The current study design and its analysis were not preregistered. Collected data will be available from Karolinska Institutet, but restrictions apply to their availability, as they were used under ethical permission for the current study and are not publicly available. However, data are available from the authors upon reasonable request and with permission from Karolinska Institutet.

### 2.6. Analysis

Qualitative content analysis (38, 39) was used to analyze the data. This method is not linked to any particular science, which reduces the risk of confusion concerning the philosophical point of departure (40). Qualitative content analysis can be used in an inductive way, which means that analytical categories and theories are generated from the empirical material. This form is often named “conventional content analysis”. A deductive form of content analysis, on the other hand, departs from already existing knowledge of a phenomenon, which is tested on the new material to confirm, expand or refine this knowledge. This form of content analysis is suggested appropriate when there is a theory or prior research about a phenomenon that can benefit from further validation or modification (38). In the current study, the authors initially adopted a deductive approach with a directed content analysis in mind, using the interview questions as a guide when reading the transcripts and make the initial coding. In the Introduction section, the authors have explained that elite athletes, according to previous research, are exposed to certain risk factors which were consequently included in the interview questions. Research on athletes' views on support to promote mental health and avoid mental disorders and health risk behaviors influenced other interview questions. Thus, there were already a theoretical framework to depart from when starting the analysis of the interviews. After performing the interviews, the researchers that had carried out the interviews met and summarized their impressions from their interviews to lay a foundation for the later systematic analysis of the transcripts. This team-based approach increases the trustworthiness of the results, since a single researchers' perspective can be questioned by the other researchers with other experiences (41). Then, one of the researchers (PK) read all the interviews repeatedly while looking for meaningful units which could be grouped into preliminary categories and codes, as exemplified in Table 2.

**Table 2:** Example of analysis.

**Meaning unit**	**Condensed meaning unit**	**Code**	**Category**
*If you take for example a larger club in a big city, then it can be extreme outbursts [in social media] and you are in and take part in it and are negatively affected by people disliking you. It is definitely a bad idea to read it. That's my opinion*.	People expressing negative opinions about players in social media negatively affects players	Social media and external performance pressure	Soccer context

The social or background information on age, living condition (household), and occupation were not included in the qualitative analysis. Apart from the phenomena connected to the interview questions, unexpected content was also considered. The authors also used manifest content analysis, in which the coder did not differ much from the informants' own expressions when analyzing the data (42). The other main form of analysis is latent content analysis and is often defined as interpreting what is hidden deep within the text (42), which was not used primarily. Staying close to the text, however, did not mean that the coder dismissed hidden messages, only that the manifest approach dominated the coding and analytical process. During the initial reading, a preliminary coding scheme with definitions (codebook) was developed (41). Because qualitative studies are at risk of different researchers drawing dissimilar conclusions from the same data, a second coder (AN) was appointed to use the preliminary coding scheme to code four interviews independently (39). At least two investigators are often involved in the coding of qualitative material to increase the trustworthiness of the results (39, 41, 43). The agreement rate between AN and PK was high, and the few disagreements on the definition of codes were solved through discussion. The coding scheme, outlined in Table 3, was presented to the whole research team, who agreed on it.

**Table 3:** Final coding scheme.

**Category**	**Soccer context**	**Health**	**Behavior**	**Attitude toward prevention**	**Prevention suggestion**
Codes	Training and playing	Physical	Alcohol	Positive	Information and dialogue
	Coach-player relationship	Mental	Other drugs	Negative	Policy and routines
	Peer-player relationship		Gambling		Individual support
	Performance pressure		Gaming		
	Norms				
	Present prevention				
	Social media and external performance pressure				

## 3. Results

The analysis generated 17 codes and the following 5 analytical categories: soccer context, health, behaviors, attitudes toward prevention, and prevention suggestions. The results are presented below, organized in accordance with the coding scheme. The brackets at the end of the quotes contain the number of the informant and indicate whether he is a junior or senior player.

### 3.1. Soccer context

#### 3.1.1. Training and playing

Several of the informants, both seniors and juniors, talked enthusiastically about playing soccer, emphasizing how fun it is.

*It's the most fun there is! Now I got this injury that will unfortunately keep me away for a while. But the only thing I wanted to hear from the doctors was, “You will be able to play soccer again,” and I will. So, my goal is just to come back and perform at as high a level as possible*. (Junior 9)

Many also suggested that soccer is the main focus in their life.

*Yes, soccer has been a part of my life since I was 3 years old. […] Before it was a little more relaxed, now it's serious. So, it's a very big part of my life*. (Junior 12)

A very experienced senior player described his feelings toward the sport during different phases of the career, emphasizing that motivation can vary during a long career.

*The deeper motivation has always been rooted in the joy of soccer and that I develop and get better and see the joy in it. Then it [the motivation] has been hidden or perhaps forgotten or influenced by other factors at different stages of my career*. (Senior 2)

#### 3.1.2. Coach-player relationship

Overall, the informants described good relationships with their coaches. The seniors especially emphasized professional relationships with good communication.

*Very good [relationship], maybe the best [coach] I've had during my career, actually. […]. We have good communication, and it feels like he understands me*. (Senior 4)

The juniors reported having had many coaches, but good relationships with most of them, emphasizing the presence of a caring approach.

*Yes, I think I have a good relationship with my coaches. I have received two new ones recently, now during the year. And yes, I feel that I have a good relationship with them. If there is something [that bothers me] I know I can turn to them and I know I can get the help I need*. (Junior 16)

Both senior and junior players, however, expressed that the coaches can be harsh and demanding. One of the junior players referred to his own performance as a factor in the relationship quality with his coach, suggesting that if he is successful, the relationship improves. Similarly, others suggested that their coaches are too focused on winning instead of communicating.

*It feels like they [the coaches] did not really understand, that they were a bit too focused on just winning and all that stuff. So, it was mostly screaming and not listening*. (Junior 17)

One of the informants emphasized that coaches' communication and behavior can influence player's mental health in a negative way, perhaps unintentionally, due to a lack of information about the player's situation.

*If you feel bad mentally, you then perform poorly in training, and it just gets worse and worse. Then you might have a coach who says something like “sharpen up,” because the coach doesn't know that this person is mentally ill. So maybe it leads to a kind of vicious circle, so that the mental strength deteriorates*. (Junior 10)

#### 3.1.3. Peer-player relationship

The relationship quality among team members appears to be good in most of the clubs. The senior players emphasized good relationships when training and playing, while junior players stated that they spend time with their teammates in their leisure time, as well, which was confirmed by a senior player who looked back on his teens.

*It [the relation to the teammates] has probably always been very good. You get very strong ties to the player squad. Then, now that you have a family, it's different. When you were single and moved to a city and only had soccer, well, then it's a completely different team bonding. You really hang out 24/7 with the players in the team*. (Senior 5)

The perception of good relationships among teammates is also indicated in the section addressing norms.

#### 3.1.4. Performance pressure

Most informants seemed to have a fairly relaxed view on the pressure to perform at a high level.

*Of course, there is some pressure on us. But I don't want to think about it. You just relax and play. It's not… if you fail you do, so does Cristiano Ronaldo*. (Junior 11)

Nevertheless, performance requirements can affect stress and sleep, especially if coaches are demanding, according to the informants. A certain performance pressure was emphasized by some of the informants as necessary to play and not sit on the bench, especially by juniors who run the risk of not being able to advance to the next level.

*Then you have to perform to be able to move on. And if you don't, then it's just “Goodbye.” So, I think it's very important to get a break from soccer sometimes*. (Junior 12)

A certain form of performance pressure (external pressure from supporters) is further described below in connection to content on the influence of social media.

#### 3.1.5. Norms

Regarding norms around health risk behaviors, players did not experience pressure to either gamble for money, drink alcohol, or use other drugs.

*No, I think it has really disappeared in recent years, that there is absolutely no such agitation in our group [to drink alcohol]*. (Senior 7)

Norms about a “healthy” lifestyle and habits with regard to for example training and diet are also communicated from the clubs, according to some informants.

Regarding openness about problems, the informants provided varied pictures. Some referred to an environment where it is not stigmatizing to talk about personal issues and stated that the clubs are aware of the importance of players' feeling free to talk about personal issues.

*We [who are older] can be open with different problems. […] It also spontaneously feels like the younger ones are quite safe in the team and can tell both players and leaders. And I think our club has been very good at that*. (Senior 7)

One of the junior players stated that he could talk about personal issues with the teammates that he felt close to.

*At least I can do it [talk about personal problems] with my closest ones in the team. Yes, I can*. (Junior 11)

Others expressed a more careful approach regarding sharing personal problems with others. One of the senior players referred to a certain “macho culture” among the players that can have an inhibiting effect around discussing problems.

*In general, it's a macho culture. There are twenty guys in a locker room, and no one wants to appear weak. So that's probably the barrier you need to pass [if you want to talk about your problems]*. (Senior 5)

The informants also expressed concern that if the coaches find out that a player has problems, it can lead to negative consequences for training and advancement.

*I think that if everyone finds out about it [gambling] in the team, and if the coaches find out, there will be consequences [for playing]*. (Junior 17)

#### 3.1.6. Present prevention

Efforts to prevent health risk behaviors and mental health problems exist to varying degrees in the clubs. Several informants mentioned that clubs host lectures where leaders or invited speakers discuss what is expected with regard to health risk behaviors, problems that can arise for individual players, and policies for such behaviors.

*It's usually a slightly larger lecture once every 2 years or once a year [about gambling, alcohol consumption and drug use]*. (Junior 10)

Some informants also mentioned policies and rules regarding behavior.

*We have an alcohol and drug policy within club [X], in the academy […]. If it were to happen, there would be consequences to it*. (Junior 9)

Several also reported that players have access to individual support when needed.

*In [club X] we have a so-called feel-good coach. A person who is there just to talk to us about... yes, about private things. And he does not share it with coaches or anything like that. So, it's a duty of confidentiality*. (Junior 19)

However, the support seems to vary among clubs. For example, larger clubs have more resources to spend on prevention activities, according to the informants.

#### 3.1.7. Social media and external performance pressure

Several informants believe that social media can result in negative pressure on the players through the disapproval that supporters who are dissatisfied with players' efforts and achievements may express. The attention that can be perceived as positive can quickly turn into the opposite if players fail or are less prominent in a game, sometimes resulting in players closing social media accounts.

*When things are going well, everyone is there. And then if you have made a shitty match, or are not in the spotlight, maybe you get a different type of response. And then you close all your accounts because you can't take it. […] I think it's a dangerous trap that is there all the time*. (Senior 2)

The importance of being able to relate to social media was also emphasized. For example, a senior player suggested that younger players may struggle more than older players to handle negative reviews and comments. Several of the younger informants, however, had positive views toward social media and explained that they also received encouraging messages from supporters.

### 3.2. Health

#### 3.2.1. Physical

The informants generally claimed to be in good physical condition.

“*I feel fit, and I feel strong and alert and happy. So, it feels good.”* (Senior 3)

Several of the players, however, have been injured during their soccer career.

*Maybe a year and a half ago, I pulled the back [of my leg] and was gone for a month and a half, 2 months. And then both my shoulders were dislocated last year*. (Junior 10)

Periods of injury can also negatively influence players' mental health, as described below.

#### 3.2.2. Mental

Most informants expressed that they are currently feeling well.

*I feel very good today. I have had previous experiences of difficult periods [concerning health], especially in my early 20s. I had one or two years there that were very difficult*. (Senior 2)

Some informants, however, expressed an existing situation of stress.

*You have your ups and downs. I think it mainly has to do with putting everyday life together. You are in so many roles. In part, you are a school student, but in the profession, you are an employee, and you are also a soccer player. In addition to that, you are a person in your spare time. So, it's hard to put it together*. (Junior 9)

Being injured for a period of time can also have a negative effect on mental health if a player does not have enough support.

*Just when I was injured, the club did not have good organization with a physiotherapist or doctor on site. And then it happened that a lot became my own responsibility. And I was 18 at the time when I had my most serious injury. And I didn't have very good experience of injuries before, so it's clear that you walked around and were a little lost*. (Senior 8)

### 3.3. Behaviors

#### 3.3.1. Alcohol

The interviews suggest that risky behaviors, including excessive alcohol consumption, are rare. Alcohol consumption appears to be increasingly uncommon among players, at least during the soccer season. Some seniors referred to earlier periods in their careers when alcohol consumption was more common; however, they stated that currently, most players want to stay in shape and are expected to take care of their physical health.

*I feel better without [alcohol], in general. For soccer, it's less advantageous. I still have an ambition to do everything I can, based on my fitness, to be able to perform and feel good. Then I know that alcohol is not really in that calculation. And I can have fun without alcohol too*. (Senior 2)

#### 3.3.2. Other drugs

If alcohol consumption is unusual among players, other drugs seem even more rare.

*I actually know nothing about that [other drugs]. […] It's very hard to believe also that it… that it is used. I would be very surprised*. (Senior 7)

One of the players stated that the soccer context does not differ from the rest of society and that drug use can occur. However, most informants had not noticed teammates taking other drugs, and one mentioned the ambition to perform well as a reason to abstain.

*It's a bit the same as alcohol, that it [other drugs] can affect soccer performance and everything. Yes, I think… Yes, it's not that good. Most people invest in the team*. (Junior 17)

#### 3.3.3. Gambling

According to the informants, gambling for money occurs to some extent among the players, but no one expressed that it is particularly problematic, at least not for themselves.

*Yes, it [occurs]. Both horse track and a little regular betting as well, but I don't know about large sums. But gambling does occur*. (Senior 3)

However, the informants suggested that there are risks with gambling and that players' access to money through high salaries can further contribute to the risk of more extensive gambling. A player who previously had problems with gambling attributes this in part to his financially favorable situation.

*Even if I had not succeeded in soccer, I still think that I would have had that interest [to gamble for money] and that I would have had problems anyway. But I had not earned as much money as I did at a young age, so it's something that comes into play*. (Senior 6)

When asked about their own economic situation, many informants reported good incomes from soccer, especially senior players. Overall, the junior players expressed a satisfactory economic situation; however, some juniors had jobs outside of football to improve their private economic situation. A player who had an acquaintance with gambling problems suggested that gambling (for money) can be based on a desire to make money, as well as excitement (sensation seeking).

*It must not be an exaggeration, of course. […] I personally have pretty good discipline when it comes to those pieces [gambling], but I don't think everyone has. And I think there are many who might chase […] some kind of kick*. (Senior 5)

Another player suggested that soccer players have excessive spare time, which can contribute to restlessness, which may result in gambling for money.

*In general, soccer players feel quite restless when not playing soccer, so to speak. There is a lot of time left over. You train in the mornings, then people go home. […] So, I think that just the time [is a factor]. And then it's clear that many people make quite good money*. (Senior 1)

#### 3.3.4. Gaming

Several informants play computer games, but this is not perceived or admitted as being problematic.

*Yes, but I'm still doing a bit of back and forth, actually. […] I would still say that I play quite a bit. I have many friends who play a lot. Not that I'm sitting inside all day playing video games*. (Junior 17)

Reasons to play computer games are, according to the informants, that they are fun and that “you get engrossed in” them. One player keeps in touch with friends in other parts of the world by playing computer games. A disadvantage of gaming that was brought forward is that relatives may think that it takes too much time.

### 3.4. Attitudes toward prevention

#### 3.4.1. Positive

Several informants gave examples of preventive measures that they perceived as good.

*You probably quite often have such lectures where an expert comes in who talks about everything. Then it's like this about match fixing, betting, and what can happen. That you can get caught up in betting and that you just want to play more to win back money and that has been quite a lot. So, I think it's good that you have lectures about it*. (Junior 13)

Several informants also had a positive attitude toward different types of policies and guidelines.

*Policies and routines or structures [to prevent mental health problems] are important if they are followed, if you work actively with them*. (Senior 2)

Furthermore, several players highlighted the positive aspect of being able to turn to an external psychologist or other professionals outside the soccer context.

*We have had a psychologist, […] We have had individual conversations with him, and he has talked to everyone about how we feel at home, mental health problems, how we feel at school, how we perform at school. […] Are you feeling well? Are you not feeling well? And it has been just me and him and you have felt that you could open up to him*. (Junior 13)

#### 3.4.2. Negative

Few negative perceptions about existing preventions were expressed by the informants. These views mainly concern modifying or improving existing efforts, including providing information of various kinds more often. Having a policy on different health risk behaviors was also perceived by several players as positive, if it was enforced.

*If you have policies just to show them, then they are not as effective. So, I think it's good to have strategies and to implement them then, so that they are not symbolic actions*. (Senior 2)

Concern about the risk of negative consequences if players admit personal problems to coaches were also expressed.

### 3.5. Prevention suggestions

#### 3.5.1. Information and dialogue

As described above, the information that is often given is perceived as positive by players, and some saw a need for more of it, including information to prevent risky behaviors. The importance of raising questions about different health risk behaviors early and on repeated occasions was also emphasized.

*Something that is good for future generations is that you invite someone at an early age who often comes and talks about such things [alcohol, other drugs, etc.] to the club. That there may be a few meetings or lectures from time to time, to give them an insight into what can happen if you start with things like this, what negative effects it has*. (Junior 16)

Despite a certain openness to talk about problems, there are also club contexts where the informants perceive that players do not dare to admit their problems. However, lectures and information may result in the destigmatization of mental health problems.

*There are probably a lot of people in our team who feel bad but don't say anything. Because it's also connected to this macho culture, that yes, you should always be strong, and so on. So I think it might be important to have lectures about it, so players understand that it's not something bad to talk about, but that there is someone there who can help them solve their problems*. (Junior 10)

#### 3.5.2. Policies and routines

Several informants also highlighted policies and routines as proposals for measures, especially if the policy is updated and enforced.

*Yes, but I actually think it would be very good, that you are very tough and have a strong policy with what should be done and what should not be done. And especially if you find out what punishments would happen, if you see that someone is abusing this*. (Junior 17)

One of the players suggested that policies and guidelines should be available on the club's website so that they are known to everyone who wants to join the club.

*Now we get to take part in it [policy]at the beginning of the seasons, but I think that during seasons we must be able to…[…] have a [web]page where there are guidelines, policies, so that you know what you agree on*. (Junior 9)

A senior player said that the players' contracts can be a tool for preventing alcohol and drug use.

*You can put different clauses or provisions in contracts, regarding consumption [of alcohol and other drugs] if you want it to be accessed in a better way. You can have that. “If you do not meet these requirements in relation to match and training”[…] this and that applies*. (Senior 2)

#### 3.5.3. Individual support

The importance of being able to talk about problems with someone was highlighted by the informants, as described above, and suggestions on how this can be promoted were provided. One example was to have a psychologist connected to the club.

*It's good if you have a psychologist in the club that you can talk to and then I think not only to a team players [but to juniors too]. [...] Because already there [at junior level], you start with performance anxiety. You want to perform. Relocations here and there and lots of that*. (Junior 13)

One of the informants also emphasized the importance of staff or leaders in the clubs telling the players where they can turn with different types of problems and to use external expertise if necessary.

*I think the club must go out and signal clearly to this team who is responsible for the different parts and who to turn to. It's not like you raise your hand in the locker room, if you have gambling problems here and now, you try to keep it for yourself maybe. […] Then I think maybe it is the club's responsibility to involve external parties in that case if you have alcohol problems for example*. (Senior 5).

Coaches' behavior has been mentioned above as an important factor affecting the players' mood. Some believe that coaches should think about their communication to improve the situation for the players.

*I actually think the coaches sometimes have a tendency to stress their players. […] They want fast results and fast performances sometimes, and that can be a problem. I think you, as a player, get a little stressed and maybe get performance anxiety or you get difficulty sleeping or whatever it may be. You may need to have a little more dialogue on an individual level, so the players feel better sometimes*. (Senior 7)

Internet-based programs or tools were also included among the proposals relating to individual support.

*Yes, I definitely think that it [internet-based interventions] can make support available to many who find it difficult to visit a psychologist physically. That it can reduce this first threshold […] If you have an app available you can turn around and talk and it can certainly be a much smaller and easier step to take*. (Senior 5)

## 4. Discussion

In summary, the informants emphasized positive feelings in relation to playing soccer and generally good health, with periods of injuries, which negatively influenced mental health, and few health risk behaviors. Elite players' large income, excessive free time, and need for excitement were identified as risk factors for problematic gambling, and experiences of stress and mental health problems were linked to performance pressure, social media, and injuries during different periods of their career. Despite the “macho culture” in the soccer context, some informants explained that players today can talk openly about personal problems with teammates and even leaders, the latter however, to a lesser extent due to a perceived risk of negative influence on advancement. The clubs' efforts to destigmatize health risk behaviors and mental health problems were put identified as promoting openness, and positive attitudes toward preventive measures were expressed.

### 4.1. Comparison with previous research

The informants in the current study shed light on several protective factors (1, 16), such as good relationships with coaches and teammates. Some players, however, explained that coaches can behave demanding manner, which may negatively influence players' mental health, especially junior players who may be stressed about not being selected for higher level teams, a finding consistent with previous studies (16, 25). Previous research further underlines the importance of coaches' awareness of how to use their power over athletes constructively and invest in their relationships with players to promote healthy personal and athletic development and increase performance (5, 35). The importance of positive relationships for players is established in previous research (16) and was recently highlighted by Sothern and Gorman (25) in a study of young English soccer players' mental health. Significant others can alleviate stigma and encourage the players to seek help for personal problems. Most of the informants in the current study lived with either a partner or parents, which may increase the opportunity to receive private support when mental health problems arise.

As indicated above, the informants in the current study testified about performance anxiety influenced by their own ambitions to advance, demanding coaches, and periods of injuries. Injuries are common at the elite level, according to previous research (1, 2, 5–10), and can negatively influence mental health (16). Gulliver et al. (44) identified similar sources for performance anxiety among young Australian elite athletes, including family members. However, family members were not mentioned as a cause of performance pressure in the present study. Instead, the impact of social media on performance pressure and performance anxiety was described as problematic. Although none of the players directly mentioned the word “shame” in relation to failed efforts and some informants underlined the positive aspects of social media, disapproval conveyed by supporters *via* social media appears to be a powerful source of stress for players. In a Norwegian study, Hofseth et al. (45) examined soccer players' different coping strategies for dealing with shame due to failures and found that both problem-focused (learning) and emotion-focused (hiding) strategies existed. Emotion-focused strategies were considered understudied at the elite level. In the current study, the authors identified emotion-focused coping in the form of closing social media accounts to avoid negative judgements from supporters. Previous research supports athletes' development of more adaptive forms of coping, such as cognitive behavioral strategies and self-management, rather than strategies to avoid negative feelings (16). Therefore, opportunities to train and develop adaptive forms of coping could preferably be provided by the clubs (16).

Health risk behaviors in the form of excessive substance use, gambling, or gaming did not appear to be prominent among the players in the current study. Previous research supports that illicit drug use is less common among elite athletes than the general population (46, 47). One of the senior informants explained that more alcohol consumption among players occurred some years ago, but this has changed. Studies among young people in Sweden and worldwide point to a general trend of decreased alcohol consumption (48, 49), which may influence soccer players, as well. The informants, however, expressed their ambition to stay fit and perform well as a reason to abstain from alcohol and illicit drugs. According to the informants, the clubs also support a healthy lifestyle. Some risk factors were suggested in relation to gambling, such as access to money, free time, and a sensation-seeking personality, which is consistent with previous research (13).

Several informants testified that players may avoid telling their coach about their problems, suggesting that acknowledging problems could have negative consequences for games and advancement (16, 25). Some informants instead highlighted the advantage of being able to talk to a specially appointed person or professional, who does not pass on information to the coach. This form of support was suggested in previous research, implying that sport organizations should consider creating confidential ways for elite athletes to discuss their mental health so that their position on the team will not be compromised based on their confidential conversations (5). Apart from the risk of missed opportunities, the stigma surrounding mental health problems was not as prominent in our study as in previous studies (44). Perhaps the various efforts to address mental health problems (10, 18, 19) among soccer players have yielded some results, although much work remains to be done to destigmatize mental health problems in the elite soccer context (5).

Apart from stigma, uncertainty about who to turn to with personal problems may prevent players from seeking support. This obstacle has also been observed in previous studies (16) and among Australian elite athletes (44). Some informants expressed appreciation for having a psychologist or other type of coach connected to the club that they could talk to, which has previously been observed as a facilitating factor when elite athletes seek help (44). This may be especially important when players get injured and cannot participate in daily training with their teams and coach.

The informants were positive about clubs regularly addressing risks associated with alcohol, illicit drugs, and gambling as a potential preventive measure. Furthermore, many informants believed that policies to prevent health risk behaviors are useful and can be effective when they are enforced. A policy to reduce illicit drug use among Australian football players, including drug testing, was previously found to be successful (20) and anti-substance use policies in other areas of society, are regarded as effective to prevent or limit substance use too (50).

Many proposals for preventive measures concerned individual external or confidential support, such as a psychologist. The internet was seen as a channel of support for players, as it is an easily accessible and anonymous way to get help, which according to previous studies lowers the threshold for addressing concerns or problems (51). In the light of this study and previous research, the support and prevention work among elite soccer players should be developed to more effectively promote health in this group (16, 25). Preparing soccer players for retirement is also important, as a number of protective factors may disappear outside the soccer context when the career is over (52).

### 4.2. Strengths and weaknesses

The current study has a number of strengths. First, we were able to recruit juniors and seniors from the total population of male elite soccer players in Sweden, which optimized the opportunity to obtain rich material, involving players with and without health risk behaviors or mental health problems. Second, several researchers participated in the collection and analysis of data, providing a form of triangulation to increase the trustworthiness of our results by decreasing the risk of confirmation bias (53). Nevertheless, this study must also be seen in the light of certain limitations. Players with more serious health or psychosocial problems may have refrained from participating, despite anonymous participation, which may mean that the results are not representative of the entire player group. Despite promises regarding anonymity, there is a risk that the informants hesitated to talk openly about problems in their teams. In addition, interview studies are always vulnerable to social desirability bias due to a potential desire to provide socially acceptable answers (54) or to avoid revealing problems, especially studies on sensitive topics among elite male athletes (55). Finally, the study is based on a relatively small number of soccer players which mean that important information may have been missed out, which in turn may limit the understanding of the studied phenomena.

## 5. Conclusion

Soccer players are exposed to a number of situations that can increase the risk of mental health problems during their career. It lies in the interest of players and clubs that players have a good mental health status so that they can perform at their best. Based on the present study, future research and implementation of interventions should focus on the prevention of health risk behaviors, alleviation of stress and performance pressure, and destigmatization of mental health problems and awareness among coaches of the importance of their communication and behavior for players' mental health and performance. This could be achieved by developing strategic and systematic policy work, information, and dialogue among players and coaches, as well as individual digital or face-to-face support, provided by professionals outside the soccer context.

## Data availability statement

The datasets presented in this article are not readily available because restrictions apply to their availability, as they were used under ethical permission for the current study. Requests to access the datasets should be directed to the authors.

## Ethics statement

The study was performed in accordance with the Declaration of Helsinki and was approved by the Ethical Review Authority (dnr. 2020-05010). Participants gave oral and written consent to participate in the study.

## Author contributions

PK: conceptualization, data curation, formal analysis, investigation, project administration, methodology, writing—original draft, and writing—review and editing. AN: data curation, formal analysis, investigation, methodology, validation, writing—original draft, and writing—review and editing. AS: conceptualization, investigation, project administration, methodology, and writing—review and editing. KB: investigation and writing—review and editing. TE: conceptualization, project administration, funding acquisition, methodology, and writing—review and editing. JG: conceptualization, supervision, funding acquisition, methodology, and writing—review and editing. All authors contributed to the article and approved the submitted version.
